# Measuring the Topological Charge of Orbital Angular Momentum Beams by Utilizing Weak Measurement Principle

**DOI:** 10.1038/s41598-019-44465-z

**Published:** 2019-05-29

**Authors:** Jing Zhu, Pei Zhang, Qichang Li, Feiran Wang, Chenhui Wang, Yingnan Zhou, Jinwen Wang, Hong Gao, Leong Chuan Kwek, Fuli Li

**Affiliations:** 1 Shaanxi Province Key Laboratory of Quantum Information and Quantum Optoelectronic Devices, School of Science, Xi’an Jiaotong University, Xi’an, 710049 China; 2 Center for Quantum Technologies, National University of Singapore, Singapore, 117543 Singapore; 3 MOE Key Laboratory for Nonequilibrium Synthesis and Modulation of Condensed Matter, School of Science, Xi’an Jiaotong University, Xi’an, 710049 China

**Keywords:** Quantum optics, Imaging and sensing

## Abstract

According to the principle of weak measurement, when coupling the orbital angular momentum (OAM) state with a well-defined pre-selected and post-selected system of a weak measurement process, there will be an indirect coupling between position and topological charge (TC) of OAM state. Based on this we propose an experiment scheme and experimentally measure the TC of OAM beams from −14 to 14 according to the weak measurement principle. After the experiment the intrinsic OAM of the beams changed very little. Weak measurement, Topological Charge, OAM beams.

## Introduction

The beams with an azimuthal phase profile of the form exp(*ilφ*) carry an orbital angular momentum (OAM) of *lℏ*^1,2^, where *φ* is the azimuthal angle and *l* is the topological charge (TC) which can be any integer value. For the infinite dimensional of *l*, the OAM beams have offered a good source both in classical and quantum optics with many different applications, such as optical tweezers and micromanipulation^3–7^, classical optical communications^8–12^, quantum cryptography^13–15^, high-dimensional quantum information^16–24^, spiral phase contrast imaging^25^, holographic ghost imaging^26^ and so on.

Prior to those applications, one of the crucial issues is the precise determination of the TC of an unknown OAM beam. Various methods have been proposed to detect the TC of OAM beams. Generally, interference is a convenient way to be employed, such as interfering the measured OAM beam with a uniform plane wave or its mirror image^27,28^. Another choice is utilizing diffraction patterns with a special mask, such as triangular aperture diffraction^29^, multiple-pinhole diffraction^30^, single or double slit(s) diffraction^31,32^, angular double slits diffraction^33–36^ and so on. Besides, geometric transformation by converting OAM state into transverse momentum states provides an efficient way to convert OAM states into transverse momentum states^37,38^. However, almost all of those methods can be regarded as a strong coupling measurement which will damage the intrinsic orbital angular momentum (OAM) of the final beam.

Alternatively, the experimental technique, weak measurement provides a feasible way to solve this problem. Proposed by Aharonov *et al*.^39^, as an extension to the standard von Neumann model of quantum measurement, weak measurement is characterized by the pre- and post-selected states of the measured system which weakly interact with the pointer system^40–42^. When the interaction is weak enough this approach will not cause state collapse. This feature makes weak measurement an ideal tool for examining the fundamentals of quantum physics, such as for the measurement of the profile of the wave function^43^, the realization of signal amplification^44^, the resolution of the Hardy paradox^45,46^, a direct measurement of density matrix of a quantum system^47^ and so on^48–51^. Recently, a theoretical method is put forward to measure the OAM state by weak measurement process with the positive integral TC of the OAM beams^52^.

## Results

In this Letter, we utilize the principle ref.^52^ proposed to measure the TC of OAM beams and extend the value of TC *l* to the negative range.

### Theoretical description

Based on ref.^52^, when the value of TC expending to the negative range an unknown OAM state in the position space can be expressed as

1 $$\langle x,y|{\psi }_{l}\rangle =\frac{\sqrt{{2}^{|l|+1}}}{\sigma \sqrt{\pi |l|!}}{(\frac{x+isgn(l)y}{\sigma })}^{|l|}\exp (-\frac{{x}^{2}+{y}^{2}}{{\sigma }^{2}}).$$

where *l* is the TC, *l* ≠ 0 and *sgn*(⋅) is the sign function. Following the scheme of weak measurement, the initial state |*φ*〉_*i*_ can be prepared as |*φ*〉_*i*_ = |*i*〉 ⊗ |*ψ*_*l*_〉, in which |*i*〉 is the preselected state. By considering the von Neumann measurement, the interaction Hamiltonian can be described as $\hat{H}=\gamma \hat{A}\otimes {\hat{P}}_{x}$, where *γ* is the coupling constant, *Â* is an observable of the preselected state and ${\hat{P}}_{x}$ is the momentum observable of the unknown OAM state. Consequently, the unitary transformation is $\hat{U}={e}^{-i\gamma \hat{A}\otimes {\hat{P}}_{x}}$. After this unitary transformation, system *Â* is post-selected to the state |*f*〉 and the unknown OAM state is projected to |*x*, *y*〉 basis. Then the final state become $\phi (x,y)=\langle f|{\langle x,y|\hat{U}|\phi \rangle }_{i}$ which contains the information about *l*. Under weak measurement *γ* ≪ *σ*, such that when $|l{|}^{2}\frac{{\gamma }^{2}}{{\sigma }^{2}}\ll 1$ is satisfied, a simplified result can be obtained as

2 $$l=-\frac{{{\rm{ReA}}}_{{\rm{w}}}}{{{\rm{ImA}}}_{{\rm{w}}}}[\frac{\bar{y}}{\bar{x}}+O({l}^{2}\frac{{\gamma }^{2}}{{\sigma }^{2}})],$$

Neglecting higher orders, we get

3 $$l\simeq {l}_{n}=-\frac{{{\rm{ReA}}}_{{\rm{w}}}}{{{\rm{ImA}}}_{{\rm{w}}}}\frac{\bar{y}}{\bar{x}},$$

where

$$\bar{x}=\frac{\int x\phi {(x,y)}^{\ast }\phi (x,y)dxdy}{\int \phi {(x,y)}^{\ast }\phi (x,y)dxdy},$$

$$\bar{y}=\frac{\int y\phi {(x,y)}^{\ast }\phi (x,y)dxdy}{\int \phi {(x,y)}^{\ast }\phi (x,y)dxdy},$$

and

$${A}_{w}=\frac{\langle f|\hat{A}|i\rangle }{\langle f|i\rangle }.$$

On the other hand, according to Eq. (28) of ref.^52^ the final state is the superposition of two OAM states with same TC which separate from initial position with the same distances of *γ* along the opposite direction. Meanwhile, the change of the intrinsic OAM which equal to the theoretical erro is very little after the measurement because of the weak interaction^53,54^.

### Experiment and result

**Figure 1:**
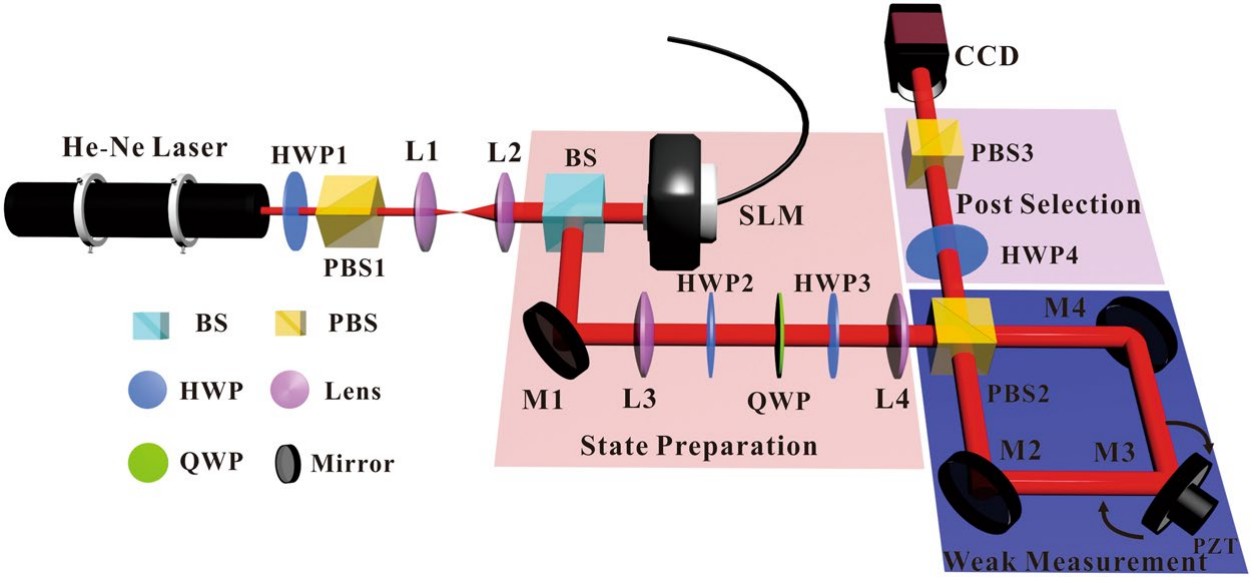
Sketch of the experimental setup.

Figure 1 shows the experimental setup of the weak measurement. Light from a He-Ne laser passes through a half-wave plate (HWP) and a polarizing beam splitter (PBS). The HWP is used to adjust the polarizing ratios and the PBS filters the desired polarization. The beam is then expanded by two lenses, L1 and L2. The expanded beam is vertically illuminated on a spatial light modulator (SLM) with the resolution of 20 *μ*m per pixel to generate OAM states. The beam then passes through two HWPs with a quarter-wave plate (QWP) inserted in-between to prepare a known polarization state as the preselected state |*i*〉. At this point, the state preparation is completed. To achieve the weak measurement operation, a polarizing Sagnac interferometer is employed in which one of the mirrors, M3, is connected with a piezo-transmitter (PZT). A single polarizing beam splitter (PBS) is used as the entry and exit gates of the device. When entering the interferometer, the incident beam is split into different polarization components |*H*〉 and |*V*〉, which traverse the interferometer in opposite directions. Without the PZT, the two components would combine again when they exit the PBS. Because of the existence of the PZT, a tiny rotation can be imposed on M3 and the two components can be separated slightly in different directions at the exit. This executes exactly the operation *Û* we mentioned above. Meanwhile the lenses, L3 and L4, constitute the 4f system which images the distribution of both *H* and *V* components after the beam is reflected by the SLM to the position of the charge-coupled device (CCD) camera. After the weak measurement, a HWP and a PBS are used to post-select the state |*f*〉. Finally, the intensity pattern is recorded by a CCD camera.

In the setup, the observable of the weak value *Â* corresponds exactly to *Â* = |*H*〉〈*H*| − |*V*〉〈*V*|. The pre-selected state is prepared as |*i*〉 = *a* exp(−*i*2*θ*)|*H*〉 + *b* exp(*iϕ*) exp(*i*2*θ*)|*V*〉 and the post-selected state is |*f*〉 = cos(2*η*)|*H*〉 + sin(2*η*)|*V*〉. The values of the parameters involved in our experiment are listed in Table 1.

**Table 1:** The values of experimental parameters.

a	b	*θ*	*ϕ*	*η*	$\frac{{{\boldsymbol{\gamma }}}^{{\bf{2}}}}{{{\boldsymbol{\sigma }}}^{{\bf{2}}}}$
0.7015	0.7126	10°	170°	18.5°	0.006

**Figure 2:**
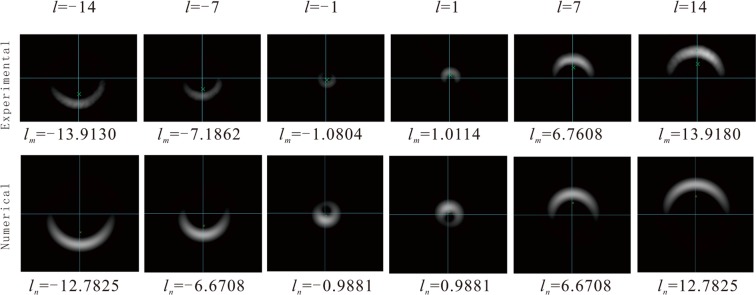
Intensity distributions of the final states for different TC of OAM beams. *l* stands for the value of the OAM initially generated. *l*_*m*_ is the experimentally measured TC of the beams and *l*_*n*_ is the numerical result. The top row is the intensity distribution of the final states in experiment while the bottom row is the simulative intensity distribution. The blue lines are the (x, y) coordinate lines and the green crosses denote centroids of the intensity distribution.

Figure 2 shows the experimental and the numerically simulated light intensity distribution of the final states. Table 2 lists the OAM of experimental and simulated beams over a range *l* = −14 to *l* = +14. where *l* represent the TC originally generated, *l*_*m*_ is the experimentally measured one and *l*_*n*_ is the result of numerical calculating.

**Table 2:** The TC values of experimental and numerical results.

*l*_*g*_	−1	−2	−3	−4	−5	−6	−7	−8	−9	−10	−11	−12	−13	−14
*l*_*m*_	−1.0804	−2.1603	−3.0953	−4.0695	−5.0895	−6.1386	−7.1862	−8.2243	−9.2286	−10.1509	−11.3784	−11.9755	−13.3031	−13.9130
*l*	−0.9881	−1.9644	−2.9289	−3.8817	−4.8229	−5.7526	−6.6708	−7.5775	−−8.4730	−9.3571	−10.2301	−11.0919	−11.9427	−12.7825
*l*_*g*_	1	2	3	4	5	6	7	8	9	10	11	12	13	14
*l*_*m*_	1.0114	1.9817	2.9786	3.9707	4.7446	5.7691	6.7608	7.9425	8.8233	10.0349	11.1854	12.357	12.8498	13.918
*l*	0.9881	1.9644	2.9289	3.8817	4.8229	5.7526	6.6708	7.5775	8.4730	9.3571	10.2301	11.0919	11.9427	12.7825

*l*_*g*_, *l*_*m*_ and *l* are as the same as Fig. 2.

## Discussion

From Fig. 1 and Table 2, it is clearly observed that the absolute value of experimental result is a little larger than the simulated one and the accuracy of the simulated value obviously decline more quickly than the experimental one with the increase of the |*l*|. This is caused by an experimental treatment. To avoid over exposure, we always adjust the parameters of the CCD to control the maximal incident light intensity. As a result, the intensity distribution recorded *I*_*m*_ is the product of the actual distribution *I* and a scaling coefficient *μ*. For the CCD has a response threshold so that the intensity below the value will not be recorded, the scale coefficient of the intensity will cause some intensity loss. Suppose that the actual light intensity is *I*(*x*, *y*) = *φ*(*x*, *y*)^*^φ(*x*, *y*) and the response threshold is *ξI*_*max*_, and then, the intensity we record in the picture (*I*_*m*_) becomes

4 $${I}_{m}(x,y)=\mu I(x,y){\rm{\Theta }}[\xi {I}_{max}-I(x,y)],$$

where $${I}_{max}=\,{\rm{\max }}\,[I(x,y)]$$, and

$${\rm{\Theta }}(t)=(\begin{array}{ll}1 & t > 0,\\ 0 & t\le 0.\end{array}$$

Replacing *φ*(*x*, *y*)^*^φ(*x*, *y*) in Eq. (3) with *I*_*m*_, we get

5 $$\begin{array}{rcl}{l}_{m} & = & -\frac{{\mathrm{Re}\underline{{\rm{A}}}}_{{\rm{w}}}}{{\mathrm{Im}\underline{{\rm{A}}}}_{{\rm{w}}}}[\frac{\int y\phi {(x,y)}^{\ast }\phi (x,y)dxdy}{\int x\phi {(x,y)}^{\ast }\phi (x,y)dxdy-\int xO(\xi I(x,y))}\\  &  & \,\,-\frac{\int yO(\xi I(x,y))}{\int x\phi {(x,y)}^{\ast }\phi (x,y)dxdy-\int xO(\xi I(x,y))}],\end{array}$$

where *O*(*ξI*(*x*, *y*)) represents the effect of the faint intensity that is not recorded by the CCD. Since the second term of Eq. (5) is much smaller than the first term, we can ignore it. It is then obvious that *l*_*m*_ is a little larger than *l*_*n*_. For the Eq. (3) is derived by neglecting the higher terms of $\frac{{\gamma }^{2}}{{\sigma }^{2}}$, the result of *l*_*n*_ is smaller than the value initially generated *l*. In this sense, Eq. (5) gives some compensation and the accuracy of the experimental results decrease more slowly. This means the restriction ${l}^{2}\frac{{\gamma }^{2}}{{\sigma }^{2}}\ll 1$ in ref.^52^ can be relaxed and the difficulty of the experiment can be reduced substantially. In addition, all the theories involved are based on the fact that *l* is an integer, so that it is reasonable to round *l*_*m*_. In this situation, our experiment is perfectly consistent with the true value.

## Method

When a state, (*a*|*H*〉 + *b*|*V*〉) ⊗ *ψ*(*x*, *y*), passes through a polarizing Sagnac interferometer with a shift of 2*γ* in linear optic system, it becomes *a*|*H*〉 ⊗ *ψ*(*x* + *γ*, *y*) + *b*|*V*〉 ⊗ *ψ*(*x* − *γ*, *y*). In addition, after the unitary transformation of $\hat{U}={e}^{-i\gamma \hat{A}\otimes \hat{P}}$ in our measuring scheme, the initial state becomes *φ*_*w*_(*x*, *y*) = (|*i*〉 + *Â*|*i*〉) ⊗ *φ*_*i*_(*x* + *γ*, *y*)/2 + (|*i*〉 − *Â*|*i*〉) ⊗ *φ*_*i*_(*x* − *γ*, *y*)/2. Let |*i*〉 = *a*|*H*〉 + *b*|*V*〉, when *Â* = *σ*_*x*_ we can get *φ*_*w*_(*x*, *y*) = (*a* + *b*)(|*H*〉 + |*V*〉) ⊗ *φ*_*i*_(*x* + *γ*, *y*)/2 + (*a* − *b*)(|*H*〉 − |*V*〉) ⊗ *φ*_*i*_(*x* − *γ*, *y*)/2. When *Â* = *σ*_*y*_, the result becomes *φ*_*w*_(*x*, *y*) = [(*a* − *b*)|*H*〉 + (*a* + *b*)|*V*〉] ⊗ *φ*_*i*_(*x* + *γ*, *y*)/2 + [(*a* + *b*)|*H*〉 − (*a* − *b*)|*V*〉] ⊗ *φ*_*i*_(*x* − *γ*, *y*)/2. And when *Â* = *σ*_*z*_, we have *φ*_*w*_(*x*, *y*) = *a*|*H*〉 ⊗ *ψ*(*x* + *γ*, *y*) + *b*|*V*〉 ⊗ *ψ*(*x* − *γ*, *y*). Obviously, apart from a polarizing Sagnac interferometer we need more optical elements to realize the weak operations for *Â* = *σ*_*x*_ and *Â* = *σ*_*y*_. This means more complicacy and experimetal error will be introduced. So we choose the experiment scheme in which a polarizing Sagnac interferometer is enough to realize the weak operation of *Â* = *σ*_*z*_.

It is important to collimate the waist radius *σ* and the coupling constant *γ* for they are the pivotal parameters to determine the theoretical error of the scheme. To collimate the beam profile we have combined two aspects. Theoretically, as we employ a 4f system, the waist radius we used must be as same as the one loaded on the SLM. Experimentally, we determine the waist radius by the following steps: (a) Record a full spot when the HWP of the post-select system is at 0° (or 45°) and calculate its centroid, *P*_*H*_ (or *P*_*V*_). (b) Choose a line passing through *P*_*H*_ (or *P*_*V*_) and retrieve the intensity along this line. The distance between the two peaks is the measured diameter of the spot in the direction of the line, named *D*_1_. (c) Choose another 3 lines which also passing through *P*_*H*_ (or *P*_*V*_) and calculate D2 to D4. The average diameter is $D={\sum }_{i=1}^{4}{D}_{i}/4$. (d) According to the relationship $\sigma =D/\sqrt{2|l|}$, the waist radius for each picture can be determined. During our experiment, all the error of *σ* between the theoretical and experimental results are less than 6%, so we still use the value of waist radius loaded on the SLM.

The shift of the two beams, |*H*〉 and |*V*〉, exiting from the polarizing Sagnac interferometer is 2*γ*. It is also necessary to determine the origin and the plus direction of the shift. To solve those problems, we can find the centroid of H beam, *P*_*H*_, when the HWP of the post-select system is at 0°. And we can determine the centroid of V beam, *P*_*V*_, when the HWP is at 45°. Then the distance between *P*_*H*_ and *P*_*V*_ is 2*γ*, the direction from *P*_*V*_ to *P*_*H*_ is the plus direction of x, the midpoint of *P*_*V*_ and *P*_*H*_ is the origin of the shift and the legnth of *P*_*V*_*P*_*H*_/2 is *γ*.
